# Retinal Cell Damage in Diabetic Retinopathy

**DOI:** 10.3390/cells12091342

**Published:** 2023-05-08

**Authors:** Jing Zhou, Bo Chen

**Affiliations:** Department of Ophthalmology, Icahn School of Medicine at Mount Sinai, New York, NY 10029, USA

**Keywords:** diabetic retinopathy, diabetes mellitus, microvascular complication, neuronal dysfunction, neurodegeneration, clinic therapy

## Abstract

Diabetic retinopathy (DR), the most common microvascular complication that occurs in diabetes mellitus (DM), is the leading cause of vision loss in working-age adults. The prevalence of diabetic retinopathy is approximately 30% of the diabetic population and untreated DR can eventually cause blindness. For decades, diabetic retinopathy was considered a microvascular complication and clinically staged by its vascular manifestations. In recent years, emerging evidence has shown that diabetic retinopathy causes early neuronal dysfunction and neurodegeneration that may precede vascular pathology and affect retinal neurons as well as glial cells. This knowledge leads to new therapeutic strategies aiming to prevent dysfunction of retinal neurons at the early stage of DR. Early detection and timely treatment to protect retinal neurons are critical to preventing visual loss in DR. This review provides an overview of DR and the structural and functional changes associated with DR, and discusses neuronal degeneration during diabetic retinopathy, the mechanisms underlying retinal neurodegeneration and microvascular complications, and perspectives on current and future clinic therapies.

## 1. An Overview of Diabetic Retinopathy

DR is a leading cause of blindness in the world ranging from working-age adults to the elderly population (20–74 years old) [1]. It is estimated that the DR population worldwide will increase from 463 million in 2019 to 578 million in 2030 and to approximately 700 million by 2045 [1,2]. A study showed that more than 30 million people (~9.4% of the US population) have diabetes, and approximately one-third of them are diagnosed with diabetic retinopathy [3]. DR patients can suffer severe vision loss if left untreated. DR is also associated with the risks of systemic vascular complications of diabetes, including stroke, cardiovascular events and heart failure [4].

Diabetes affects all cells in the retina, though most studies have focused mainly on retinal microvascular pathology. Based on the presence of neovascularization, DR is classified into two stages, non-proliferative (NPDR) and proliferative diabetic retinopathy (PDR) [5,6,7]. NPDR is an early stage of DR. Early morphological signs of NPDR include basal membrane thickening, tight junction impairment, and blood–retina barrier (BRB) breakdown. Moreover, there is a cell loss of pericytes and endothelial dysfunction, resulting in fragile capillaries, formation of microaneurysms, small hemorrhages, cotton-wool spots, and capillary non-perfusion. These vascular lesions accumulate to induce ischemic conditions in some areas of the retina; as ischemia develops, proangiogenic factors such as vascular endothelial growth factor (VEGF) release, inducing the formation of neovascularization, a hallmark of proliferative DR [8]. The proliferative stage of DR is characterized by retinal neovascularization due to ischemia and hypoxia. Newly formed blood vessels are relatively fragile and susceptible to retinal and vitreous hemorrhage, leading to tractional retinal detachment and vision loss. Diabetic macular edema (DME) is characterized by macula thickening induced by abnormal accumulation of extravascular fluid in the macular, and hard exudates can occur at any stage [9]. The prevalence of DME in patients with DR is 2.7–11% [10,11], and it depends on the type of diabetes and duration of the disease. DME and PDR are the main reasons for vision loss in patients with diabetic retinopathy and are increasing in prevalence around the world.

Several animal models of diabetes have been developed to study the pathogenesis of DR and to test therapies. These animal models were generated via genetic manipulation or induction. The most commonly used genetic models include db/db (Leprdb), Ins2^Akita^, non-obese diabetic (NOD), Insulin2Q104del (Kuma), and Akimba mice; and the induced animal models include administration of drugs such as streptozotocin (STZ) and alloxan, applying laser or chemical damage to the eye, feeding high galactose diet, and surgical removal of the pancreas [12,13,14,15,16]. STZ administration is the most commonly used induced model as it results in the fastest rate of disease development. Neuronal apoptosis and reactive gliosis are the common histological features in the retina with DR. The complete picture for the DR disease mechanisms and the sequence of cellular events in the DR development is far from understood because each of the animal models can only mimic certain aspects of DR pathology in human patients. We will discuss in more detail various aspects of retinal cell damage in studies using some of these animal models.

## 2. Diabetic Retinopathy and Retinal Vasculature

**Figure 1:**
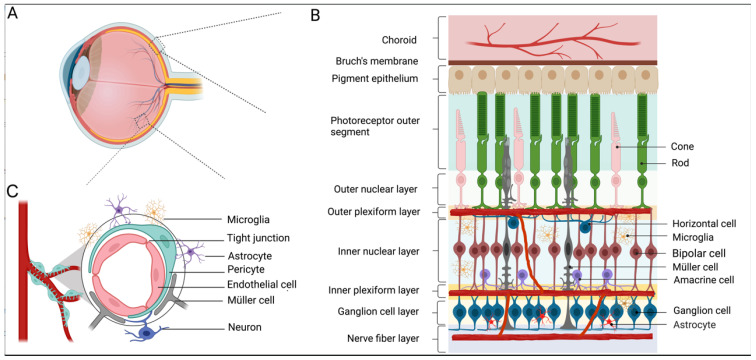
Retinal structure, iBRB, and neurovascular unit. (**A**,**B**) A drawing of a human eye with a schematic enlargement of the retinal structure. The retina lines the back of the eye, which consists of six types of neurons from the outer to the inner retina: rod/cone photoreceptors, interneurons (bipolar cells, horizontal cells, and amacrine cells), and ganglion cells as well as three types of glial cells (Müller cells, astrocytes, and microglia). The central retinal artery enters the eye through the optic nerve and branches into three vascular plexuses: superficial vascular plexus, intermediate vascular plexus, and deep vascular plexus localized in the ganglion cell layer, inner plexiform layer, and outer plexiform layer, respectively. (**C**) A schematic of the neurovascular unit and the inner blood–retina barrier (iBRB). iBRB is formed by tight junctions of adjacent endothelial cells. Pericytes, glial cells, and neurons surrounding the retinal vessel all together form a neurovascular unit to maintain the barrier function.

The retina is a specialized tissue for light detection and vision formation, composed of several neuronal cell types (rods, cones, horizontal cells, bipolar cells, amacrine cells, and retinal ganglion cells) and glial cell types (Müller cells, astrocytes, and microglia). RGCs (retinal ganglion cells) are the sole output neurons located in the most inner layer of the retina, and they relay processed visual information from the retina and the brain for perception (Figure 1A,B).

The retina relies on a well-functioning blood supply to maintain its functionality. The blood supply to the retina comes from two distinct vascular plexuses: the choroid that lines under the retina pigment epithelium to supply 90% of the total blood to the outer retina and the intraretinal blood vessel that primarily supports the inner retina [17,18]. The choroid blood vessels consist of fenestrated endothelial cells, whereas intraretinal blood vessels consist of non-fenestrated endothelia cells surrounded by pericytes, making the inner retinal vasculature form the blood–retina barrier (BRB) that regulates molecular traffic between the blood and the retina [19] (Figure 1B,C).

As one of the most energy-demanding tissues, the retina needs sufficient nutrients and high levels of oxygen to maintain its visual function [18,20]. The central retinal artery enters the eye through the optic nerve center and branches into superficial, intermediate, and deep layers. The highly specialized vasculature, together with neurons and glia, is integrated to form the retinal neurovascular unit to maintain homeostasis and modulate neuronal activities [20]. The components of the neurovascular unit of the retina include neurons (RGCs, bipolar cells, amacrine cells, and horizontal cells), glia (Müller cells, astrocytes, and microglia), and vascular cells (endothelial cells and pericytes) [21,22,23] (Figure 1C). Neurovascular coupling is the process by which neural activity is linked to the blood flow and metabolism, allowing the retina to regulate the blood flow in response to neural activities or metabolic demands [24]. All components of the neurovascular unit work closely to maintain the integrity of the inner BRB and dynamically coordinate the local blood flow in response to metabolic demands. Retinal blood vessels dilate when flickering light stimulates them, whereas breathing 100% oxygen causes them to constrict. Retinal pathology is likely to involve all these components at varying degrees. Evidence shows that complications in diabetes impair the normal function of the retinal neurovascular unit. In the pathogenesis of DR, neurodegeneration and glial activation are widely observed, even occurring before the clinical signs of DR appear, in the experimental models of DR and from the retinas of diabetic donors [22].

BRB consists of two compartments: outer BRB (oBRB) is composed of retinal pigment epithelial cells, representing a highly selective barrier for molecules and solutes moving from the choroid into the retina, and inner BRB (iBRB), which is formed by adherents and tight junctions between adjacent retina capillary endothelia cells. iBRB, established by tight junctions between endothelial cells that are surrounded by pericytes and glial cells (Müller cells, astrocytes, and microglia), plays an important role in regulating the microenvironment and thus is crucial for proper vision. Distinctive characteristics of iBRB including tight junctions and lack of fenestrations in iBRB make it a highly selective barrier in nature. iBRB controls the transport of molecules, ions, and water between the vascular lumen and the retina. The transporters in iBRB play an essential role through the release of trophic factors and antioxidants into the retinal microenvironment [25]. Damage to the tight junctions of the iBRB causes disruption of the integrity of this inner barrier [26]. iBRB breakdown is a hallmark of diabetic retinopathy [27,28,29].

## 3. Neurodegeneration in Diabetic Retinopathy

For many years, diabetic retinopathy has traditionally been regarded as a microvascular disease and the current diagnosis of DR indeed relies on vascular alterations. However, emerging evidence suggests that retinal neurodegeneration is also involved in DR, even before clinical signs of DR appear [30]. During the early stage of diabetes, several morphological and electrophysiological alterations occur in neurons of the inner retina [26,27,28] before vascular changes, leading to the recommendation that precautions against DR should be taken immediately after diabetes is diagnosed. A deeper understanding of neurodegeneration during DR is essential for early detection and targeted therapies to prevent vision loss.

### 3.1. RGCs and DR

RGCs, located in the inner retina, are likely to be easily damaged because they are highly active in metabolism, making them particularly vulnerable to local and systemic metabolic stressors. Indeed, RGCs are the neurons in which the apoptotic process related to diabetes is first detected. The death of RGCs and the degeneration of the inner nuclear layer (INL) in the postmortem eye of people with diabetes were observed early back in 1961 [31]. Supporting the clinical data, experimental evidence also showed apparent RGC loss in diabetic rat models [32,33,34]. The first quantitative report of an increase in neural cell apoptosis in the diabetic rat retina shows that RGC degeneration is observed at the early stage after the onset of diabetes, even before the degeneration of retinal capillaries in diabetes [35], postulating that neurodegeneration may contribute to capillary degeneration. In the three-month streptozotocin (STZ)-induced diabetic rat, loss of RGCs is associated with morphological change; remaining RGCs in the diabetic retina, especially those in the RGA group (a subtype of RGCs, with a large soma and a large dendritic field), show significant enlargement of the dendritic field [36]. In STZ-induced diabetic rats, there are reduced axon numbers and diameters at the distal potion of the optic nerve at the early stage of diabetes mellitus without observing morphological changes of RGCs, which indicates the optic nerve is the early structural change in the diabetic visual pathway [37]. Studies in other species such as diabetic dogs show animals under moderate glycemic control have appreciable vascular alterations without apparent degeneration of RGCs [38].

Although neurodegeneration in RGCs in mice is less apparent compared to that in rats, the time-dependent loss of RGCs has been observed in many of the DR mouse models [39,40]. Yang et al. reported the apoptosis of RGCs in db/db mice, a model of spontaneous type 2 diabetes, whereas there was no obvious abnormality in the retinal vasculature [41]. RGC apoptosis and cell loss were also detected in Ins2^Akita^ mice within the first 3 months of retinal hyperglycemia, together with a reduced IPL thickness after 5.5 months with other marked alterations in the morphology of the surviving cells. In addition to the RGC loss, abnormal morphologic swellings were observed in the dendrites and axons, such as enlargement of the RGC soma and the extent and density of dendritic branches in ON-type RGCs [42,43,44]. In STZ-induced diabetic mice, RGC loss begins in 6~12 weeks after induction through apoptotic cell death. There is approximately a 30% RGC loss based on the studies of STZ-induced diabetic models [45,46]. In particular, the RGC complex, including the nerve fiber layer, the ganglion cell layer, and the inner plexiform layer, is affected, which is accompanied by a significant decrease in the density of RGCs and amacrine cells [47]. In STZ-induced diabetic mice, the morphology and passive membrane properties of the dendrites in ON-type RGCs are preferentially affected at the early stage, which is RGC subtype-dependent [48]. Interestingly, diabetes accelerates the longitudinal RGC dysfunction independently of elevated intraocular pressure, as evidenced in a combined model of STZ-induced diabetes in the DBA/2J model of glaucoma; diabetes exacerbates early progression of glaucomatous RGC dysfunction between 3 and 6 months of age, without influencing the intraocular pressure [49]. Nonetheless, other studies did not observe an obvious loss of RGCs in the STZ-induced models, likely due to different reactions of STZ in different animal strains.

Morphological changes in RGCs together with physiological alterations will influence how they process signals from the photoreceptors to the brain. A recent study showed an early response of RGCs in diabetic retinopathy, involving specific morpho-functional deficits in most RGC subtypes but there was no RGC loss [50]. Neuronal protective treatment using a chemical somatostatin analogue octreotide preserves the functionality of RGCs, highlighting the importance of neuronal protection in the early phase of diabetic retinopathy; RGC morphology can be preserved or adjusted to maintain RGC physiology [50]. This provides new therapeutic hopes for patients in the early phase of diabetic retinopathy.

### 3.2. Müller Cells and DR

Müller cells are the primary support glial cells in the retina. Müller cells span the entire thickness of the retina, come into contact with all types of retinal cells, and wrap around blood vessels, making Müller cells functionally unique and significant in maintaining retinal homeostasis. Müller cells express various voltage-gated channels and neurotransmitter receptors, which enable them to modulate neuronal activity by regulating the extracellular concentration of neuroactive substances, including K^+^, glutamate, GABA, and H^+^ [51]. In addition to supporting the structure and function of the retina, Müller cells also play an important role in the vascular function of the retina. The presence of Müller cells, astrocytes, and microglia, together with pericytes wrapping around the blood vessels, are all essential to maintain the homeostasis of the retina [29]. In the retina, astrocytes are restricted to the nerve fiber layer and RGCs in the vitreous side of the retina, and Müller cells play a predominant role in interacting with astrocytes and RGCs. Recent studies using diabetic animal models indicate that Müller cells are involved in dysfunctional aspects in diabetes by influencing BRB, especially at the early DR stage. Using a conditional Müller cell ablation model, a study shows that Müller glial deficiency may cause neuronal and vascular pathologies in retinal diseases [52]. Based on these diabetic animal models, depletion of Müller glia results in BRB breakdown and vascular alterations, hallmarks of DR pathology, indicating that Müller dysfunction may be a primary contributor to the vasculopathies of DR.

Reactive gliosis is one of the first responses to the inflammation in DR and is characterized by upregulation of various kinds of molecules, including the most well-established GFAP [53,54]. In the healthy retina, GFAP is mostly detected in astrocytes, with relatively low expression in Müller cells. GFAP activation is a key feature of any kind of impairment of the retina, such as mechanical damage, light damage, and injuries in various retinal diseases. In the STZ-induced diabetic rats, reactive changes in Müller cells, such as upregulation of GFAP, are observed within 3~4 months of the onset of diabetes while GFAP immunoreactivity in astrocytes is reduced, all of which precede the first signs of obvious vascular changes [55,56]. Another study pays particular attention to the first 4 weeks after STZ injection; induction of hyperglycemia occurs within 2 weeks and Müller cells undergo hyperplasia before GFAP expression, indicating that glial cells are the early targets of vascular hyperpermeability in the diabetic retina. In addition, microglia, the resident immune cells in the retina, also undergo highly dynamic morphological and functional alterations [54].

In addition, Müller cells produce high levels of pro-inflammatory molecules and neurotoxic factors, resulting in reactive gliosis under hyperglycemic conditions [31]. Single-cell transcriptomic analysis from DR shows that Müller cell subpopulation genes are involved in the lysosomal pathways, lysosomal membrane permeabilization, and leakage of lysosomal contents, all of which are known to lead to cell death [57]. It is noteworthy that DNA damage response 1 (REDD1) [58] is specifically expressed in Müller cells with the same expression pattern as Müller markers, contributing to diabetes-induced retinal pathology; REDD1 knockout mice exhibit reduced oxidative stress with no obvious retinal thinning or neurodegeneration in the RGC layer, providing a potential clinical therapeutic target for future DR therapy [58].

### 3.3. Photoreceptors and DR

Photoreceptors are the most abundant cells in the retina that convert light into neural signals. The photoreceptors and the retina pigment epithelium (RPE) in the outer retina normally function as a unit to maintain proper visual function. Phototransduction in photoreceptors involves a sequence of enzymatic reactions, including conversion of 11-cis retinal to all-trans retinal, causing the key conformational changes and activation of G proteins, followed by hydrolysis of cGMP to GMP, closure of cGMP-gated ion channels, hyperpolarization, and less glutamate release from these cells. RPE works with photoreceptor cells to regenerate 11-cis retinal through the classical visual cycle to support the function of both rods and cones [59,60].

To better understand the correlation between photoreceptors and DR, we need to understand O_2_ distribution and usage in photoreceptors and the retina. The demand for energy in the retina is exceedingly high and much of the energy is derived from oxidative metabolism coupled with ATP synthesis, which is mainly dependent on continuous supply of oxygen [59,61,62]. The oxygen that is supplied to the retina cannot be stored in any region of the retina and must be in the vascular and choroidal vascular circulations of the retina. Photoreceptors use more energy at night when photoreceptor ion channels are open than in the daytime. In darkness, the surfaces of the outer segments of the rods are depolarized and leaky; water and sodium entering the retina must be extruded by pumps in the inner segment and thus more energy and oxygen are consumed within the inner segments to support ion pumping [63]. Therefore, photoreceptor activity in the dark makes the retina more hypoxic than the normal condition; this is especially the case for retinal vasculature diseases such as DR. Indeed, reduced or loss of dark adaptation is the first symptom in a variety of pathological conditions [64,65,66].

Growing evidence suggests that photoreceptors are affected in DR. Early studies on the association between poor color vison and signs of retinopathy date back to 1972 and 1973, indicating that blue-yellow and blue-green vision losses were much more severe in Scottish diabetic patients than normal controls [67,68]. Subsequently, other studies showed impairment of color vision, specifically blue-sensitive defects [69,70], as well as decreased contrast sensitivity in DR patients [71,72,73]. The rate of apoptosis increases in the outer nuclear layer with a reduction in photoreceptors between 4 and 24 weeks after the onset of diabetes [73]. Nevertheless, diabetes has not been reported to cause widespread degeneration of photoreceptors in patients and animal models. Up to now, conflicting results remain regarding whether DR induces photoreceptor death [74].

While emerging evidence shows that photoreceptors may be impacted by DR, many studies support that photoreceptors contribute to the development of retinal vascular lesions and early characteristics of DR by releasing inflammatory proteins [75,76,77,78]. Accumulation of inflammatory proteins in the diabetic retina accelerates the development of vascular lesions; when the production of proinflammatory proteins is inhibited, vascular lesions are also inhibited [79,80]. Using a model of retinitis pigmentosa (in particular rhodopsin knockout mice) in which the diabetic environment is induced via STZ, Gooyer et al. show that loss of the outer retina reduces the severity of diabetic retinopathy due to decreased hypoxia [78]. Photoreceptors are the main source of reactive oxygen species in the retina; deletion of photoreceptors inhibits the diabetes-induced increase in superoxide [75]. Photoreceptor cells themselves produce various proinflammatory and inflammatory proteins, such as interleukin-1β (IL-1β) [81], interleukin-1α(IL-1α), chemokine C-X-C motif ligand 1(CXCL1), monocyte chemoattractant protein 1(MCP-1), CXCL12a, chemokine ligand 25 (CCL25), TNF-a [76], inducible nitric oxide synthase (iNOS), intercellular adhesion molecule-1 (ICAM1), and vascular endothelial growth factor (VEGF) [75,78]. Some of these proteins have been reported to increase endothelial permeability or alter the tight junction and cell adhesion proteins [10,76,82].

## 4. Molecular Mechanisms Underlying Neurodegeneration in DR

Diabetic retinopathy was originally considered a microvascular disease; increasing evidence shows that it is a chronic inflammatory disease that leads to changes in the microcirculation of the retina. This raises a question whether the inflammation processs is involved in diabetic retinopathy; anti-inflammatory drugs should be able to alleviate various aspects of DR. In 1964, a study on a patient with rheumatoid arthritis showed a high regression rate of diabetic retinopathy as the patient had been taking a high dose of aspirin for 12 years [83]. Another study administered aminoguanidine and aspirin on a daily basis for 5 years in a dog model of DR, and both treatments were found to effectively inhibit the development of acellular capillaries in diabetes [84], indicating that inflammation is associated with DR. Using cDNA arrays to examine gene expression patterns in the diabetic retina in STZ-induced diabetic rats, upregulation of the inflammatory components was detected during the early stage of the disease onset [85]. DR is the manifestation of a mild chronic inflammation, during which inflammatory effectors including cytokines, pro-apoptotic molecules, and leukocytes are released and responsible for damages to the vascular endothelium of the retina. This type of damage is slow and cumulative over time. As diabetes progresses, acellular capillary forms and irreversible ischemia develops, leading to the discharge of certain vasoactive chemicals such as VEGF that promote the formation of new blood vessels and the transition to the proliferative stage of DR.

Hyperglycemia leads to considerable metabolic abnormalities. It causes non-enzymatic glycosylation as a result of the formation of complex cross-linked substances known as advanced glycation end products (AGEs). AGEs are a major consequence of sustained hyperglycemia during diabetes, which can trigger secondary complications. For example, increased levels of intracellular reactive oxygen species (ROS) [86,87] inflict oxidative damage to the retina. Multiple signaling pathways may have been altered in DR that include the polyol pathway [88], the protein kinase C pathway [89], and the protein kinase (MAP kinase) pathway [90], as well as abnormal activity of nuclear factors such as highly activated nuclear factor-*κ*B (NF-*κ*B). NF-*κ*B can be activated via VEGF and translocated to the nucleus to promote the transcription and expression of VEGF itself; in the meantime, it also induces the expression of other pro-inflammatory mediators such as ICAM-1, vascular cell adhesion molecule-1 (VCAM-1), monocyte chemotactic proteins 1 (MCP-1), and cyclooxygenase-2 [91]. ICAM-1, VCAM-1, and VEGF in turn are implicated in BRB disruption that causes microaneurysms and leakage in the retina [92]. Nuclear factor erythroid 2-related factor 2 (Nrf2) is one of the primary regulators of cellular redox homeostasis, which controls the transcription of downstream antioxidant enzymes but its transcriptional activity is impaired in DR [93].

Oxidative stress is an imbalance between excessive generation of ROS and their removal [82]. ROS are oxidant molecules that contain an extra electron conferring on them great instability and reactivity. Common forms of ROS include hydrogen peroxide (H_2_O_2_), peroxyl radical (ROO·), superoxide anion (O_2_·^−^), hydroxyl radical (·HO), and nitric oxide (NO·) [94]. Oxidative stress induces inflammation and mitochondrial dysfunction, leading to cell death through pyroptosis, apoptosis, or autophagy, and the resultant neurodegeneration causes neural vascular and retinal tissue damage. Oxidative stress is a critical contributor to the pathogenesis of diabetic retinopathy and also results from metabolic abnormalities induced by hyperglycemia. As we mentioned earlier, the retina is a highly energy-demanding organ and therefore is highly vulnerable to and easily damaged by high levels of ROS. Oxidative stress in DR has the ability to act as a trigger, modulator, and the link within the complex web of pathological events that occur in DR, including activation of protein kinase C (PKC), the hexosamine biosynthetic pathway, as well as the presence of increased amounts of AGEs and activation of receptors for AGE (RAGE).

## 5. Future Perspectives

Pathophysiological mechanisms underlying diabetic retinopathy are complex. Conventional treatment is based on vitreoretinal surgery and laser photocoagulation; however, traditional surgical treatment is only for proliferative diabetic retinopathy cases with hemorrhage or tractional retinal detachment. Drug treatment has been an emerging therapy to treat DR such as anti-VEGF or steroid drugs, which also target end-stages of the disease after damage has already occurred. Leading clinical anti-VEGF drugs including ranibizumab, bevacizumab, and aflibercept, have been widely utilized to treat DR patients [95,96,97]. Early anti-VEGF injection, before complications of DR have developed, can reduce further progression into severe stages [98]. However, anti-VEGF therapy shows low efficacy in certain patient populations [91]. A new technology of comparative ligandomics has identified Secretogranin III (Scg3) as a novel disease-associated ligand that selectively binds to the diabetic vessels but not healthy vessels to induce angiogenesis, and thus Scg3 may provide a new therapeutic target for antiangiogenic therapy of DR [99,100]. Although much effort has been made to investigate DR, currently there are no therapeutic strategies that can fully reverse the retinal damage caused by DR.

Neurodegeneration is an early event in diabetic retinopathy; therefore, one possible and targeted therapeutic strategy is to prevent or slow down neurodegeneration in the early stage of DR. The use of neuroprotective substances holds great potential for the treatment of DR. Endogenous neuroprotective agents, including insulin-like growth factor 1 [5], pigment epithelium-derived factor (PEDF) [101], somatostatin (SST), pituitary adenylate-cyclase-activating polypeptide (PACAP), glucagon-like peptide-1 (GLP-1), and neurotrophins such as brain-derived neurotrophic factor (BDNF) and nerve growth factor (NGF), are potential neuroprotective factors for DR [39,102]. Peroxisome proliferator-activator receptor alpha (PPARα) has been identified as a putative therapeutic target for retinopathy in type 1 and 2 diabetes [103,104]. Fenofibrate, a PPARα agonist, manifests unprecedented neuroprotective effects in DR with type 1 diabetes [103]. Further studies are needed to examine the protection and maintenance of RGC morphology and physiology. The contribution of neurotrophic factors and VEGF to diabetic RGC degeneration is controversial [105], as BDNF neuroprotection in the retina is concentration-dependent. Inadequate amounts of BDNF promote neuroretinal apoptosis and degeneration [106]. VEGF has neuroprotective effects at the early stage of DR, while at the late phase of DR, VEGF promotes diabetic neovascularization [105].

Current diagnosis for neurodegeneration in diabetic retinopathy can be measured by frequency domain optical coherence tomography (FD-OCT), allowing for detecting morphological changes such as thinning of the ganglion cell layer as well as the nerve fiber layer. Measurements of these two parameters using OCT provide valuable information about the extent of neurodegeneration in DR, representing the most practical way to monitor neurodegeneration. Functional abnormalities of retinal neurons at the early stage of DR can be measured via mfERG [107], standard automated perimetry, frequency doubling perimetry, or microperimetry, among which mfERG is the gold standard.

Gene therapy is a potential therapeutic strategy for DR and is designed to deliver genetic materials to patients or animal models for therapeutic purposes. Adeno-associated viruses (AAVs) have become the most popular gene therapy tool for the treatment of ocular disease for several of their advantages, including minimal toxicity, lower immune responses, and the ability to sustain long-term treatment effects. Although many unanswered questions remain, a number of AAV serotypes have been tested, among which AAV serotype 2 is best characterized and has been commonly used for gene therapy in humans [108]. In 2017, an AAV-based gene therapy was approved for the human-inherited retinal disease, RPE65 mutation-associated Leber’s congenital amaurosis (LCA), through delivering a functional copy of RPE65 cDNA to retinal pigment epithelial cells (RPEs). This is a historic landmark revolutionizing the therapies for retinal degenerative diseases [109,110]. Current possible gene therapy for DR mainly targets two pathological aspects: existing retinal neovascularization in the late phase of DR and neurodegeneration in the early phase of DR. VEGF is a prominent therapeutic target for DR. Various treatments have been attempted to interfere with VEGF pathways using AAVs, such as soluble VEGF receptor-1 (sFlt-1), Flt23k, an intraceptor inhibitor of VEGF containing VEGF-binding domains 2-3 of Flt-1, to suppress retinal neovascularization in animal models [101,111,112,113]. Erythropoietin (EPO) [114], a hematopoietic cytokine produced in the fetal liver and adult kidney, was reported to have a potent neuroprotective effect in the retina. AAV2-mediated delivery of EPO via subretinal injection was attempted in mouse diabetic retinas with reduced breakdown of the blood–retina barrier and less neuronal apoptosis in the outer nuclear layer [114]. Gene therapy holds promise to provide long-term treatment effects for DR, among many other desirable features compared to traditional drugs and surgical therapies. Clinical trial barriers are mainly due to the complexity of DR pathogenesis, as well as gene delivery safety and targeting.

## Data Availability

Not applicable.
